# Retained Placenta

**Published:** 1862-06

**Authors:** F. R. Payne

**Affiliations:** Marshall, Ill.


### CHICAGO MEDICAL EXAMINER.

N. S. DAVIS, M.D., Editor.

VOL. III. JUNE, 1862. NO. 6.

### Original Contributions.

### ARTICLE XVI.

### RETAINED PLACENTA.

By F. R. PAYNE, M.D., of Marshall, Ill.

Read before the Æsculapian Society, at Paris, Ill., May 28, 1862.

All practitioners deeply feel the responsibility that rests upon them in the third stage of labor. Nearly all of our best obstetrical writers regard this as the trying hour, not only of the mother and child, but also the practitioner. Ignorance at this hour may result in the loss of the mother and child, and bring reproach upon the physician. Fully impressed with the importance of the subject, I propose briefly to discuss, and knowing that my positions are not fully endorsed by many of our obstetrical instructors, it is expected and desired that the members of this society enter into a full and honest discussion of the subject. We all know that the practical departments of our profession are not perfect, consequently we love to read the experience of our professional brethren, when we know that it is honestly and fairly given. It would be infamous quackery, if not downright murder, for a physician to hatch up cases to support a favorite theory. Experience is worth nothing unless it is intelligently, honestly, and truthfully given.

Before I proceed to present my cases and experience relating to retained placenta, it would perhaps be proper to state that there is no direct communication between the vessels of the fœtus and those of the mother. The fœtal tufts are simply bathed in the maternal blood, and draw nourishment by their own cells from the mother. The placenta is truly the absorbing and respiratory organ of the fœtus.

In a practice of nearly twenty years, and the management of over one thousand labors, I have met with three cases in which retention of the placenta could not be overcome.

Case 1.—In 1854, I visited Mrs. L. aged 36 years; she had a pretty good constitution, and was the mother of six children. The confinement prior to this, there was considerable difficulty in removing the secundines. She lived six miles from town, and had employed Mrs. Rhodes, a midwife living in the neighborhood. The child was born without serious difficulty, but the woman in attendance could not get the placenta. I was sent for, and arrived at the house about ten hours after the birth of the child. In a few moments a vaginal examination was made, and I soon learned that the placenta could not be reached without the introduction of the hand into the cavity of the uterus. A large anodyne was given, and the patient permitted to rest until its narcotic effects were visible. My hand was then well oiled, and a slow, cautious effort made to introduce it into the womb. This operation produced much pain, the parts were swollen and tender, but I finally reached the cavity of the uterus, and learned that the placenta was on the right side, near the fundus, and partially adherent. A slow, gentle effort was made to detach it, the patient complained bitterly. About one-third of the afterbirth was in my hand. At this stage, the patient was seized with severe rigors, and in a few moments had a violent convulsion. The hand was withdrawn, bringing with it that portion of the placenta that was detached. The cord and all of the membranes were left in the uterus. The following was directed:—

Hyd. sub. muri............................15 grs.
Pul. Opium,.............................. 6 grs.

Make three powders, one every three hours, if no operation in five hours salts and senna. Also, the free use of uva ursi tea.

After this, I used vegetable cathartics every twenty-four hours, and continued the opium powders in two to three grain doses.

She remained unconscious for four days, and on the eighth day, the remaining secundines were expelled, and the woman rapidly recovered. She has given birth to a healthy child since that time, and now enjoys good health.

There were no bad effects made manifest by the absorption of decomposed matter—the system was not poisoned, neither was there alarming hemorrhage. In this case two-thirds of a full grown afterbirth was expelled in eight days by nature, without injury to the mother.

CASE 2.—Mrs. B. aged 33 years, aborted during the fifth month. The child was expelled ten or twelve hours before I was called. A vaginal examination revealed the fact that the os uteri was firmly and rigidly contracted, and the secundines were in the womb. A large anodyne was given, and in a few hours I made a long, persevering effort to remove the placenta. Every effort to dilate the os brought from the patient unmistakable evidence of great agony. After two hours of untiring perseverence, I resolved to leave the case to nature. Ordered pul. opii. every four or five hours, and the free use of uva ursi tea. Her bowels were kept regular by mild cathartics. In eight days, the secundines were expelled from the womb, and the patient rapidly recovered. It may be proper to state, that I made several unsuccessful efforts to remore the offending substance before nature came to her relief. The lady got along well; the room "and adjoining apartments" were not "filled with an intolerable stench." She had no more hemorrhage than in ordinary cases, and no evidence of the absorption of decomposed matter into the system; and has since this accident given birth to a healthy child.

CASE 3.—Mrs. A. The husband of this lady, a very intelligent and worthy man, called at my office, and stated that his wife had slight labor pains. She was five or six months advanced in pregnancy—first child. She was able to move about the house; general health good. I supposed the pains were caused by dilitation and expansion of cervex uteri; ordered perfect quiet and gave a few powders of sulp. morphia.

The next day I was called to see her, and learned that she had suffered severely during the first part of the night with labor pains, but for eight or ten hours they had entirely ceased. There had been a discharge of liquor amonii and slight hemorrhage. An examination revealed a child in the vagina—shoulder presentation, right hand in the womb. Without much difficulty or pain I brought the head down and delivered a dead child. After this I took the cord in my left hand and made it slightly tense, and with my right index finger followed it to the os uteri, which I found irresistibly rigid. The cord was firmly grasped by the os, and I could not introduce my finger into the womb.

A large dose of morphia was given, and after its effects were made manifest, a prolonged and cautious effort was made to dilate the os and remove secundines, but without success. The finger finally entered the os, but my patient suffered greatly; the placenta was distinctly felt but could not be removed. Thinking the usual instruments invented for dilating the os not superior to my finger, further effort was not made, and the case was left to nature. On the ninth day she had slight pains, and the secundines were expelled. Up to this time she was quite well and cheerful: felt like she could get up and move about the house, but when the pains came on that threw off the placenta—she had considerable hemorrhage. This soon ceased, and the lady rapidly recovered and now enjoys good health.

With these cases, briefly reported, the question arises, what are the causes of retained placenta? Prof. MILLER gives three: atony of the uterus; morbid adhesion of the placenta; irregular contractions of the uterus.

Atony of the womb may readily be detected. The uterus is large, flabby, and shapeless; there are no pains, considerable hemorrhage, and the afterbirth cannot be reached with the finger. Protracted labors are liable to result in uterine atony. BAUDELOCQUE thinks a too rapid expulsion of the fœtus may cause atony. Dr. DEWEES says, retention from want of tonic power must not be interfered with until by friction the organ is compelled to contract under the hand. Prof. MILLER does not like this rule, from the fact that friction over the region of the womb will not always make it contract; but he then knows he has a *hand.* He also contends that the introduction of the hand into the cavity of the uterus, in cases of atony, is "neither a painful nor difficult operation." This is all true if the accoucheur is on hand when the child is born.

But if, as is frequently the case in country practice, ten or twelve hours elapse before you reach the patient, the operation is not only painful and difficult, but liable to result in serious injury to the patient. Prof. MILLER says, "Let it be remembered, then, that the placenta cannot be allowed to remain in the womb without the imminent risk of alarming hemorrhage, which may occur at any moment, and destroy the patient before the practitioner can come to her rescue." He also states, "if let alone it *might* do well." Dr. BLUNDELL remarks, that he has "noted more than one case in which the placenta had remained for a long time in the uterus without a single conspicuous symptom of irritation becoming manifest."

I do not quote these authorities for the purpose of discouraging at all times the introduction of the hand into the cavity of the uterus when it is necessary and can be done without serious injury to the patient, it is certainly right, and the physician would surely fail in the proper discharge of his duty if, after all other means fail, he forgets that he has a *hand.*

RETENTION FROM MORBID ADHESION.—This is not a common cause of retention, but when it does occur, more danger is to be apprehended than in simple atony. I have never had a case of complete morbid adhesion. In case one, it was partial, but the hemorrhage was not alarming. If I should meet with such cases, the recommendation of BOUDELOQUE and DEWEES will not be followed,—that is force to the placenta, by means of the cord, for the purpose of disrupting its attachments. This is surely unsafe and might result in inversion of the uterus. Dr. MILLER says, "the existence of morbid adhesion may be *suspected* when the uterus feels firmly contracted and the placenta is, notwithstanding, so high as to be beyond the reach of the finger, but it can only be certainly detected by the hand carried into the cavity of the uterus."

RETENTION FROM IRREGULAR CONTRACTIONS OF UTERUS.—In relation to this form of retention, I cannot speak from experience. A case has never occurred in my practice in which I could detect, according to the rules laid down by Prof. MILLER and other obstetrical writers, irregular contraction of the womb. But I have found, in two cases of abortion after quickening, irresistible rigidity of the os, at the same time the uterus forming a hard regular ball in abdomen. This normal contraction of the womb always banishes my fear of excessive hemorrhage. Dr. RAMSBOTHAM says, "We hear much of hour-glass contraction of the uterus, but my belief is, there are no rarer cases in midwifery than the real and true hour-glass contraction." This accords with my observation.

Dr. D. O. PRIESTLEY recently read a paper on retained placenta, before the Obstetric Society of London, in which he says, "In the third, fourth, and fifth months the decidual cavity is obliterated, the placenta has acquired more intimate attachment to the womb, and the contractile power of the uterus being greater, rupture of the membranes commonly takes place before expulsion. The embryo having escaped, the secundines may soon follow, or lying in the os uteri they may be removed by the finger or some simple instrument. In a considerable number of abortions, however, in which the fœtus and liquor amnii have been voided the secundines are not soon extruded, and after the uterus has made repeated ineffectual efforts to expel them the os uteri closes and action ceases." We occasionally meet with cases of this kind, and they are exceedingly embarrassing from the fact that our obstetrical instructors differ widely as to the treatment we ought to adopt when the secundines are not spontaneously expelled from the womb. Dr. DENMAN, DAVIS, RAMSBOTHAM, and DEWEES are positively opposed to any attempt at extraction by the hand, and Dr. INGLEBY says, "No manual extraction can be effected prior to the sixth month." Drs. BURNS and CHURCHILL think manual interference in such cases is only occasionally allowable. Dr. TYLER SMITH recommends the forcible removal of the secundines in all cases of abortion when they are retained. He says he has met with a considerable number of patients who seriously suffered from placental retention. His dangers as stated from retained secundines are as follows:—

1*st.* Flooding of a serious character, and the patient being liable to hemorrhage so long as the secundines remain in the womb.

2*d.* Absorption of decomposed secundines, local inflammation of the uterus and surrounding tissues, and poisoning of the system.

3*d.* Sub-involution of the uterus.

4*th.* Generation of morbid growths and hemorrhage following their expulsion. We do not doubt that bad effects may result from retained placenta.

But the question is, if the os is intensely rigid and a protracted, painful effort to dilate it accomplishes nothing, what shall the accoucheur do?

My answer would be: let it alone and your patient will be more likely to get well than she would if you resort to extraordinary manual interference. It has been my fixed purpose since I entered the profession, not to kill my patients by an extraordinary heroic resort to remedies.

I will state, not for the purpose of boasting of superior skill, (which I do not claim) that out of over one thousand cases of labor under my charge only three have died from any cause resulting from or connected with the parturient state. One of these cases was a dwarf, whose pelvis had not sufficient capacity to give birth to a full grown fœtus, and was called too late to successfully perform the cesarean operation.

The other was a hearty, fat primipara patient. She had 29 convulsions before the child was born, and died in about ten hours after the womb had been cleared.

The third was a strong, healthy, short-necked, muscular woman, *primipara.* The labor was ushered in by violent pain in the anterior portion of the cerebrum. She lived six miles in the country. When I reached there I learned she had passed through 13 convulsions. Her skin was dark; pulse quick; and every symptom indicated approaching dissolution. At my request, Dr. C. L. Duncan was called, and my forceps were also sent for. The os was dilated barely sufficient to learn that it was a vertex presentation. My treatment was virtually that recommended by Dr. Bedford, in his "Principles and Practice of Obstetrics," (a book every practitioner ought to have.) She had 42 violent convulsions; and about two hours after the birth of the child she died. I shall, on some future occasion, report this case in full. This paper is prepared and presented to the Society for the purpose of eliciting discussion, and I hope the older members especially will speak freely on the subject I have thus briefly introduced.

